# hvTRA, a novel TRAIL receptor agonist, induces apoptosis and sustained growth retardation in melanoma

**DOI:** 10.1038/cddiscovery.2016.81

**Published:** 2016-12-12

**Authors:** Karianne G Fleten, Vivi Ann Flørenes, Lina Prasmickaite, Oliver Hill, Jaromir Sykora, Gunhild M Mælandsmo, Birgit Engesæter

**Affiliations:** 1 Department of Tumor Biology, Oslo University Hospital, The Norwegian Radium Hospital, Oslo, Norway; 2 Department of Pathology, Oslo University Hospital, The Norwegian Radium Hospital, Oslo, Norway; 3 Apogenix GmbH, Im Neuenheimer Feld, Heidelberg, Germany; 4 Department of Pharmacy, Faculty of Health Sciences, University of Tromsø, Tromsø, Norway

## Abstract

In recent years, new treatment options for malignant melanoma patients have enhanced the overall survival for selected patients. Despite new hope, most melanoma patients still relapse with drug-resistant tumors or experience intrinsic resistance to the therapy. Therefore, novel treatment modalities beneficial for subgroups of patients are needed. TRAIL receptor agonists have been suggested as promising candidates for use in cancer treatment as they preferentially induce apoptosis in cancer cells. Unfortunately, the first generation of TRAIL receptor agonists showed poor clinical efficacy. hvTRA is a second-generation TRAIL receptor agonist with improved composition giving increased potency, and in the present study, we showed hvTRA-induced activation of apoptosis leading to an efficient and sustained reduction in melanoma cell growth in cell lines and xenograft models. Furthermore, the potential of hvTRA in a clinical setting was demonstrated by showing efficacy on tumor cells harvested from melanoma patients with lymph node metastasis in an *ex vivo* drug sensitivity assay. Inhibition of mutated BRAF has been shown to regulate proteins in the intrinsic apoptotic pathway, making the cells more susceptible for apoptosis induction. In an attempt to increase the efficacy of hvTRA, combination treatment with the mutated BRAF inhibitor vemurafenib was investigated. A synergistic effect by the combination was observed for several cell lines *in vitro*, and an initial cytotoxic effect was observed *in vivo*. Unfortunately, the initial increased reduction in tumor growth compared with hvTRA mono treatment was not sustained, and this was related to downregulation of the DR5 level by vemurafenib. Altogether, the presented data imply that hvTRA efficiently induce apoptosis and growth delay in melanoma models and patient material, and the potential of this TRAIL receptor agonist should be further evaluated for treatment of subgroups of melanoma patients.

## Introduction

Malignant melanoma is a highly metastatic disease with poor survival rate. Despite recent advancements leading to novel treatment options, such as the mutated BRAF inhibitor, vemurafenib and the immune activator, ipilimumab, there is still no curative treatment for the majority of patients with advanced disease.^1,2^ New therapeutic options are therefore of great importance in order to improve clinical outcomes. TRAIL receptor agonists (TRAs) have been suggested as promising anticancer candidates as they preferentially induce apoptosis in tumor cells, while normal cells are generally unaffected.^3,4^ TRAs induce apoptosis by binding to Death Receptor 4 (DR4/TRAIL receptor-1) or Death Receptor 5 (DR5/TRAIL receptor-2), leading to receptor clustering and activation of the extrinsic apoptotic pathway.^5^ In contrast to the promising results obtained in preclinical models, all clinical trials trying to establish TRAs as drugs for human use have failed so far.^6–13^ The reasons for the observed clinical failures of the first-generation TRAs are related to short *in vivo* exposure of the drug due to its fast elimination,^11^ insufficient multimerization efficacy *in vivo*,^14^ low expression of the TRAIL-receptors DR4 or DR5,^15,16^ loss or incomplete activation of pro-caspases 3 and 8^15,17,18^ and upregulation of the anti-apoptotic protein Bcl-xL.^19^ Despite the negative clinical outcomes, there are still activities ongoing to develop TRAs for clinical use due to the potential benefits for patients. One second-generation development is represented by the recently described synthetic fusion protein APG350 (hvTRA).^20^ It consists of two trivalent single-chain TRAIL receptor-binding domains, which are covalently linked to each other by a silenced IgG1-Fc domain, resulting in a hexavalent TRAIL receptor agonist (hvTRA). Due to its unique molecular layout, hvTRA facilitates close-proximity multimerization of DR4/DR5 and thereby induces efficient activation of intratumoral apoptosis that is independent of Fc*γ* receptor-driven secondary crosslinking events *in vivo*. Consequently, hvTRA has been proven to be more efficient than other TRAs in preclinical models of various cancer types.^20^

Combining therapies, through cooperative inhibition or stimulation of multiple targets, offer a promising approach for effective treatment, and different drugs have been combined with TRAs in attempts to increase the efficacy of the drugs.^21–25^ Approximately 50% of all melanoma patients harbor mutated BRAF causing constitutive active MAPK/ERK pathway. Inhibition of the MAPK/ERK pathway can influence survival by affecting proteins in the intrinsic apoptotic pathway such as Bim, BMF and Bad,^26–29^ and reduce the stability of the anti-apoptotic protein Mcl-1,^27^ thus priming the cells for apoptosis induction.^30,31^

MAPK/ERK pathway inhibition together with TRAs is a potentially beneficial combination that stimulates both the extrinsic and intrinsic apoptotic pathways. However, conflicting *in vitro* results for the combination have been reported.^21,32^

The aims of this study were to investigate the efficacy of hvTRA alone and in combination with the mutated BRAF inhibitor vemurafenib in melanoma cell lines, xenograft model and patient materials. Our results show that hvTRA effectively reduce the viability of melanoma cells both *in vitro* and *in vivo* and strongly encourage further evaluation of hvTRA alone. However, vemurafenib-induced downregulation of DR5 seems to represent a limiting factor for therapeutic success of combining hvTRA and vemurafenib.

## Results

### hvTRA reduce melanoma cell viability, lung tissue colonization and tumor growth

**Figure 1:**
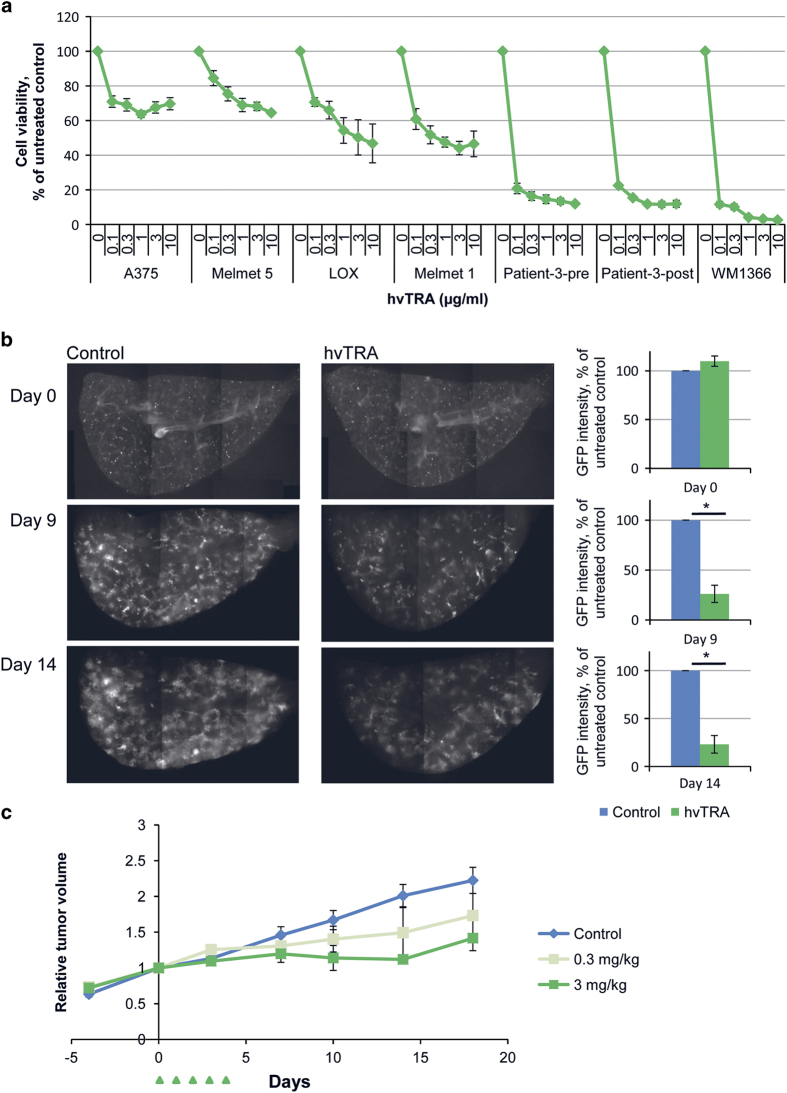
hvTRA reduced cell viability in melanoma cells and initiate the extrinsic apoptotic pathway. (**a**) Seven melanoma cell lines were exposed to increasing concentrations of hvTRA. Cell viability was measured after 72 h by the MTS assay. The experiments were repeated three times and error bars represent±S.E.M. (**b**) Left panel: representative pictures of the pulmonary metastasis assay (PuMA) showing growth of Melmet 5-GFP cells *ex vivo* in lung tissue exposed to hvTRA (0.25 *μ*g/ml) at day 0, 9 and 14. Right panel: quantification of fluorescence from the Melmet 5-GFP cells in the lung tissue section using ImageJ. The fluorescent intensity of the control samples was set to 100% for each time point, and the intensity of hvTRA-treated samples were related to the control from the same day. Four biological replicates were performed with three to four technical replicates. (**c**) Melmet 5 xenografts grown subcutaneously in nude mice were treated with 0.3 or 3 mg/kg hvTRA (i.v. injection) as indicated in the treatment schedule depicted in the figure by 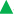. The tumors were measured twice a week using a caliper, and are presented as relative tumor volume related to the volume of the tumor at the initiation of the treatment. Three mice (six tumors) were included in the control group, and two mice (four tumors) were included in both groups treated with hvTRA. Error bars represent±S.E.M. *P<0.05.

The potential of hvTRA to reduce cell viability was examined in seven melanoma cell lines. As shown in Figure 1a, all cell lines demonstrated a dose-dependent reduction in viability after treatment with hvTRA for 72 h. The strongest response was observed in Patient-3-pre, Patient-3-post and WM1366, whereas A375 and Melmet 5 showed the least responsiveness. Cleavage of pro-caspases 3 and 8, Bid and PARP indicate that hvTRA induce apoptosis through the extrinsic apoptotic pathway (Supplementary Figure 1).

As melanomas often metastasize to the lungs, colonization of lung tissue slices in the *ex vivo* PUMA assay^33^ was used to further explore the efficacy of hvTRA. hvTRA significantly reduced the growth of Melmet 5-GFP-Luc cells measured by decreased GFP fluorescent intensity compared with the intensity in untreated lung slices on days 9 and 14 (Figure 1b). The efficacy of hvTRA was also evaluated *in vivo*, where Melmet 5 xenografts were treated with two different concentrations of hvTRA (0.3 or 3 mg/kg) for 5 days. Similar to the *in vitro* experiments, a dose-dependent reduction in tumor volume was observed (Figure 1c). In summary, the results suggest that hvTRA inhibits melanoma cell viability in various models *in vitro*, *ex vivo* and *in vivo*.

### hvTRA and vemurafenib induce synergistic effects *in vitro*

**Figure 2:**
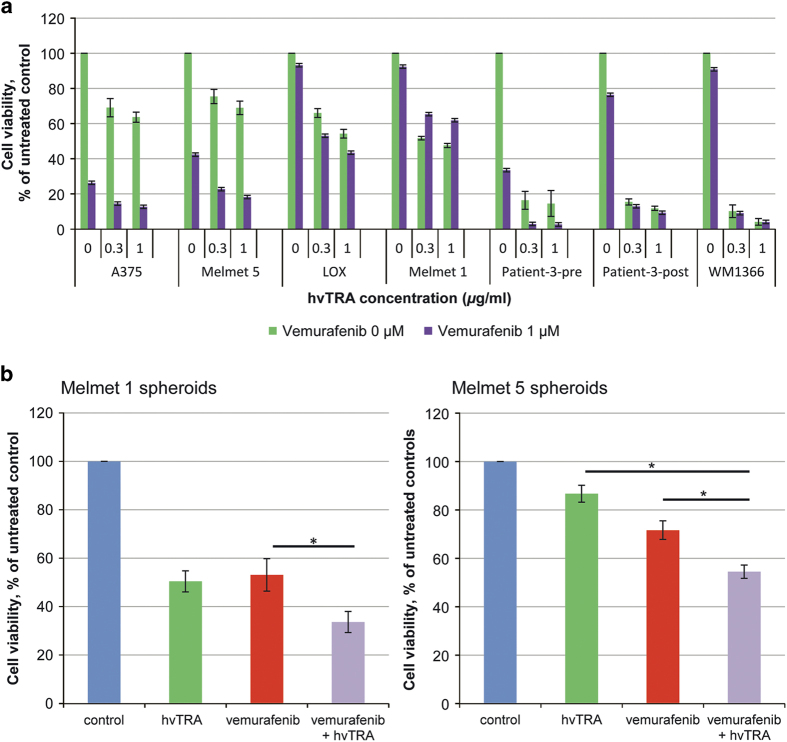
*In vitro* response to hvTRA, vemurafenib and the combination. (**a**) Seven melanoma cell lines were exposed to the combination of vemurafenib (1 *μ*M) and hvTRA at selected concentrations (purple bars). The data representing hvTRA mono treatments (green bars) are the same results as shown in Figure 1a. Cell viability was measured after 72 h by MTS assay. The experiments were repeated three times. (**b**) Melmet 1 and Melmet 5 were grown as spheroids and treated with hvTRA (0.5 *μ*g/ml), vemurafenib (0.3 *μ*M) or the combination. Cell viability was measured using CellTiter-Glo Luminescent assay and measured 96 h after start of treatment. At least four biological replicated were performed, with four technical replicates. The error bars represent±S.E.M. *P<0.05.

Previous studies have shown increased efficacy of TRAs when combined with inhibitors of mutated BRAF.^21,34^ In this study, the combination hvTRA and the clinically used BRAF inhibitor, vemurafenib, was examined. The effect of vemurafenib alone was established in selected cell lines. A strong dose-dependent response was observed in A375, Melmet 5 and Patient-3-pre, whereas in LOX, Melmet 1, Patient-3-post and the wild-type BRAF cell line WM1366 the response was modest (Supplementary Figure 2). Subsequently, we exposed the cells to hvTRA (0.3 and 1 *μ*g/ml) in combination with 0.3 (data not shown) and 1 *μ*M vemurafenib (Figure 2a). Potential synergy was evaluated by calculating the combination index (CI) using the Calcusyn software (Supplementary Table 1). The combination had a synergistic effect in A375, Melmet 5, LOX, Patient-3-pre and Patient-3-post cells, whereas an antagonistic effect was observed in Melmet 1.

Compared with monolayer cultures, spheroids are supposed to better reflect the *in vivo* conditions with respect to drug efficacy.^35,36^ When Melmet 1 spheroids were treated with hvTRA and vemurafenib alone and in combination, we found, in contrast to the monolayer cultures, that the drug combination was more effective than hvTRA (*P*=0.091) and vemurafenib (*P*=0.045) alone (Figure 2b). Likewise, Melmet 5 spheroids were significantly more responsive to the combined treatment than to hvTRA (*P*<0.001) and vemurafenib (*P*=0.028) alone.

### Combining hvTRA and vemurafenib gives an increased initial antitumor effect *in vivo*

**Figure 3:**
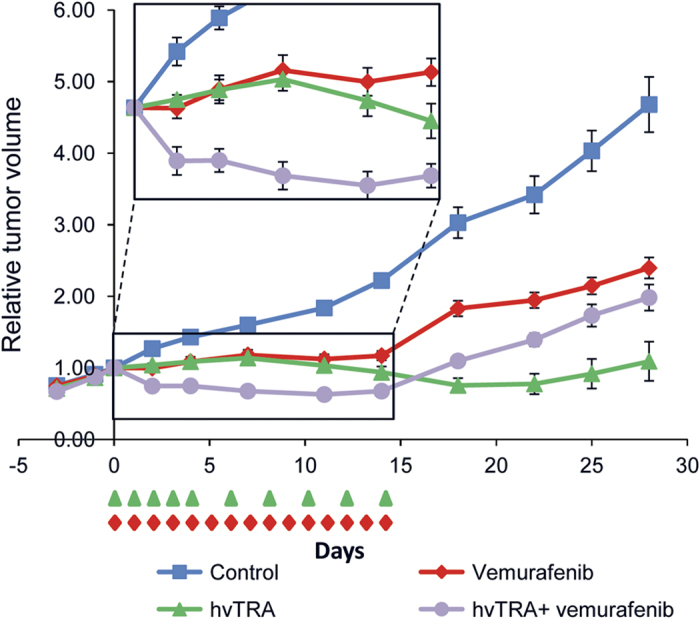
The antitumor effect of hvTRA, vemurafenib and the combination *in vivo*. Melmet 5 xenografts were treated with hvTRA (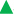) (3 mg/kg, i.v. injection) or vemurafenib (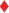) 50 mg/kg, orally twice a day) or the combination of hvTRA and vemurafenib (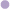). The treatment schedule is indicated in the figure by 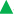 and 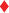. The tumors were measured twice or thrice a week using a caliper, and are presented as relative tumor volume related to the volume of the tumor at the initiation of the treatment. Eight tumors are included in each group. Error bars represent ±S.E.M.

Altogether, our *in vitro* results suggest that combining hvTRA with vemurafenib could be effective at least in a subset of melanomas. To evaluate the efficacy *in vivo,* the growth of Melmet 5 xenografts were followed while treating the mice with hvTRA (3 mg/kg), vemurafenib (50 mg/kg) or the combination as indicated in Figure 3. Both mono treatments inhibited the growth of Melmet 5 xenografts. Xenografts treated with vemurafenib remained stable during the treatment period, while, notably, a regression in size was observed in xenografts treated with hvTRA after day 10. Xenografts treated with both hvTRA and vemurafenib experienced a strong initial decrease in tumor size, not observed for any of the mono treatments, and the tumor volumes were significantly reduced compared with the tumors exposed to any of the mono treatments or controls from days 2 to 11 (insert Figure 3*; P*<0.005). The tumor volume in the vemurafenib and combination groups increased after seponation, while interestingly, the tumors treated with hvTRA did not start growing again until 8 days after seponation (day 25).

### Regulation of DR5 expression by hvTRA and vemurafenib

**Figure 4:**
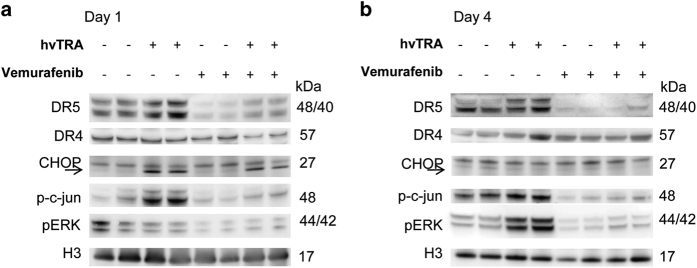
Evaluation of apoptotic proteins in response to hvTRA, vemurafenib or the combination measured by western immunoblot. Western immunoblot detection of DR5, DR4, CHOP, p-c-jun and pERK to investigate the Melmet 5 xenografts response to hvTRA and vemurafenib after (**a**) 1 day or (**b**) 4 days of treatment with vemurafenib (50 mg/kg twice a day), hvTRA (3 mg/kg) or the combination. CHOP is indicated by an arrow. H3 was used as loading control. Tumors were harvested 2 h after the last treatment. Each lane in **a** and **b** represents one individual tumor.

Both DR4 and DR5 are expressed in Melmet 5 xenografts (Figure 4), but DR4 may not be functional as response to a DR4 agonist *in vitro* is lacking (data not shown). DR4 expression in the xenografts seems not to be affected by any of the treatment regimens, whereas DR5 levels were upregulated after 1 and 4 days of hvTRA treatment and strongly downregulated in xenografts treated with vemurafenib or the combination (Figures 4a and b). Downregulation of DR5 by vemurafenib was also observed *in vitro*, but only using higher concentrations than utilized in the combination experiments (data not shown). The transcription factor CHOP and phosphorylation of the transcription factor c-jun positively regulates the transcription of the *DR5* gene.^37^ Our data showed increased phosphorylation of c-jun in tumors receiving only hvTRA both on days 1 and 4, corresponding to the observed upregulated DR5 level in the same samples (Figures 4a and b). However, p-c-jun was dephosphorylated in response to vemurafenib treatment. Expression of CHOP was exclusively observed in samples treated with hvTRA, both alone and in combination on day 1. The level of CHOP and phosphorylation of c-jun can be influenced by MAPK/ERK pathway signaling,^37^ and the presented data correspond to the activity of the signaling pathway. Reduced levels of pERK1/2 were observed in response to vemurafenib, indicating reduced MAPK/ERK activity.^38^ Interestingly, hvTRA increased pERK1/2 levels on day 4 and also slightly counteracted the downregulation of pERK1/2 induced by the BRAF inhibitor on days 1 and 4 (Figures 4a and b).

### Regulation of apoptosis-related proteins by hvTRA and vemurafenib

**Figure 5:**
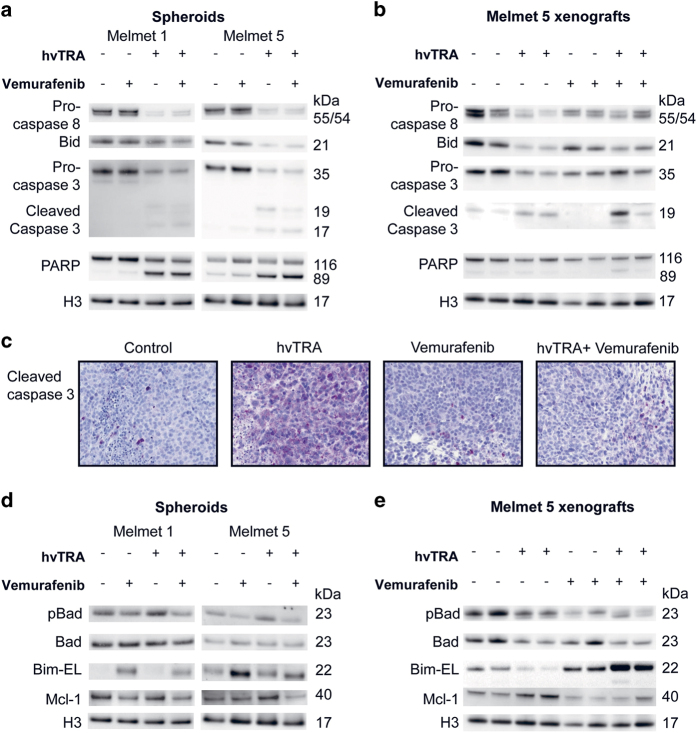
Evaluation of proteins in the extrinsic and intrinsic apoptotic pathways in response to hvTRA and vemurafenib treatment. Western immunoblot of proteins in the (**a**) extrinsic and (**d**) intrinsic apoptotic pathways in Melmet 1 and Melmet 5 spheroids in response to hvTRA (0.5 *μ*g/ml), vemurafenib (0.3 *μ*M) or the combination after 24 h of treatment. Two biological parallels of the spheroid experiments were performed. The results presented in (**a** and **d**) are from the same cell lysate. H3 was used as loading control. (**b**) Western immunoblot of proteins in the extrinsic and (**e**) intrinsic apoptotic pathway in Melmet 5 xenografts treated with hvTRA (3 mg/kg), vemurafenib (50 mg/kg twice a day) or the combination. Each lane in **b** and **e** represents one individual tumor. The same cell lysate is used in **b** and **e**. H3 was used as loading control. (**c**) Immunohistochemical detection of cleaved caspase 3 in Melmet 5 xenografts treated with hvTRA (3 mg/kg), vemurafenib (50 mg/kg twice a day) or the combination. Tumors were harvested on days 4, 2 h after the last treatment.

As demonstrated for monolayer cultures, hvTRA initiated the extrinsic caspase cascade also in melanoma spheroids (Figure 5a) and xenografts (Figure 5b). This was shown by cleavage of pro-caspase 8 and Bid. Notably, no differences in the protein levels were observed between spheroids treated only with hvTRA and the combination. However, xenografts harvested after hvTRA treatment for 4 days demonstrated more cleavage of pro-caspase 8 and Bid compared with xenografts treated with both hvTRA and vemurafenib (Figure 5b), suggesting reduced intratumoral apoptosis in the latter group. Cleavage of PARP was only observed clearly in spheroids (Figure 5a), while in xenografts the level of cleaved PARP in hvTRA-treated samples was similar to control (Figure 5b). The two xenografts treated with combination therapy showed conflicting results as one xenograft showed higher degree of PARP and pro-caspase 3 cleavage compared with xenografts treated with hvTRA only, while the other showed comparable levels. Cleavage of caspase 3 (Figures 5a and b) was not observed in spheroids or xenografts treated with vemurafenib, indicating that apoptosis is not executed in response to vemurafenib. The level of cleaved caspase 3 in the xenografts was also investigated by IHC (Figure 5c). The staining intensity of cleaved caspase 3 was highest following hvTRA alone, replicating the results observed by western immunoblotting, suggesting that vemurafenib inhibits the efficacy of hvTRA.

In an attempt to unravel the molecular effects of the various treatments in more detail, proteins in the intrinsic apoptotic pathway were studied. As shown in Figures 5d and e, dephosphorylation of pro-apoptosis Bad^ser112^ protein, an indication of activation of the intrinsic apoptotic pathway, was seen following exposure to vemurafenib in both spheroids and xenografts, while Bad was downregulated in response to hvTRA in xenografts. The pro-apoptotic protein Bim-EL was downregulated in hvTRA-treated and upregulated in vemurafenib-treated xenografts. Following combined treatment, the Bim-EL protein level was further increased compared with vemurafenib alone *in vivo* (Figure 5e), while *in vitro* Bim-EL level was reduced after combination therapy compared with vemurafenib alone. The anti-apoptotic protein Mcl-1 was downregulated in Melmet 5 spheroids after the combined treatment, while in Melmet 1 spheroids and Melmet 5 xenografts the Mcl-1 level was decreased both after treatment with vemurafenib alone and in combination. hvTRA increased the Mcl-1 levels *in vivo*.

### Reduced vemurafenib dosing does not give a synergistic long-term effect

**Figure 6:**
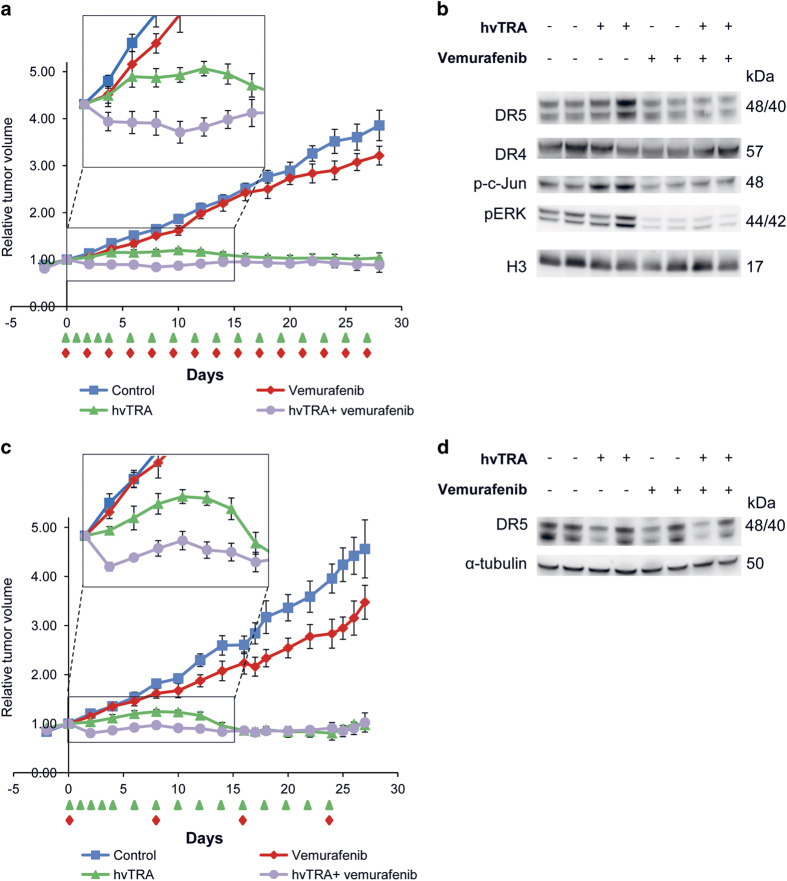
Growth reducing capacity of hvTRA, vemurafenib and the combination with reduced concentrations of vemurafenib. Melmet 5 xenografts were treated with hvTRA (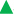) (3 mg/kg), vemurafenib (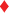) ((**a**) 50 mg/kg every second day) or (**c**) 50 mg/kg twice a day every 8th days) or the combination of hvTRA and vemurafenib (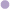). The treatment schedule is indicated in the figure by 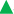 and 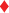. The tumors were measured twice or thrice a week using a caliper, and are presented as relative tumor volume related to the tumor volume at the initiation of the treatment. Eight tumors are included in each group in **a**, and five tumors in each group in **c**. Error bars represent±S.E.M. (**b**) Western immunoblot detection of DR5, DR4, p-c-jun and pERK in response to hvTRA and vemurafenib treatment (50 mg/kg every second day). Tumors were harvested on day 4, 2 h after the last treatment. H3 was used as loading control. (**d**) Western immunoblot detection of DR5 in response to hvTRA and vemurafenib treatment (50 mg/kg twice a day every 8th days). Tumors were harvested 8 days after the last treatment. *α*-tubulin was used as loading control. Each lane in **b** and **d** represents one individual tumor.

Our previous *in vivo* study revealed that hvTRA significantly reduces tumor growth, and combining hvTRA with vemurafenib gave no additional long-term effect. The initial cytotoxic effect observed in the combination group in Figure 3 suggests a potential advantage of the combination. The transient response may however be explained by vemurafenib-induced repression of the DR5 expression (Figure 4). It was therefore of interest to adjust the vemurafenib treatment regime in an attempt to maximize the cytotoxic response and minimize the effect on the TRAIL receptor. Reducing the vemurafenib dose to one-fourth (50 mg/kg once a day every second day) resulted in no tumor growth delay in the vemurafenib-treated group (Figure 6a). Xenografts receiving combination treatment showed initial decrease in tumor size, but not as pronounced as with the higher vemurafenib dose. After the initial decrease, the tumor size remained stable. There was a significant difference in tumor volume between xenografts treated with hvTRA and the combination from day 4 to day 10 (*P*<0.05). In the vemurafenib and the combination-treated xenografts, the DR5 level was slightly lower compared with the control (Figure 6b). p-c-jun followed the same trend as DR5 with the highest level in hvTRA-treated samples, and a weak reduction in vemurafenib-treated samples compared with the control. When the frequency of vemurafenib dosing was reduced, the cleavage of Bid and pro-caspases 3 and 8 was similar between hvTRA alone and the combination (Supplementary Figure 3A), which could indicate that hvTRA was able to bind to DR5 and activate the extrinsic apoptotic pathway. However, the concentration of vemurafenib has been too low to give an additional inhibition of tumor growth compared to hvTRA mono treatment.

A slight reduction in DR5 expression was still observed in response to the reduced dosing frequency of vemurafenib (Figure 6b). Therefore, another *in vivo* experiment was initiated with the intention that DR5 expression would be completely restored between each time vemurafenib was given. Vemurafenib (50 mg/kg) was given twice a day every 8th days, while hvTRA dosing was kept as before (Figure 6c). hvTRA showed similar effect as previously with regression in tumor size after day 10. As observed in the preceding experiments, there was an initial decrease in tumor volume in response to the combination. The subsequent vemurafenib treatment on day 8 induced a slight decrease in tumor volume, while later administrations on days 16 and 24 gave no additional effect. The size of the tumors treated with hvTRA or the combination were reduced to a similar level. DR5 levels were investigated 8 days after the last treatment. No regulation of DR5 were observed in response to vemurafenib, indicating that 8 days are enough for the DR5 levels to return to normal (Figure 6d). In conclusion, our results indicate that combining hvTRA and vemurafenib did not enhance the effect of hvTRA, while hvTRA as mono treatment induced a stable and sustained growth delay in all experiments performed.

### hvTRA can reduce cell viability in patient biopsies

**Figure 7:**
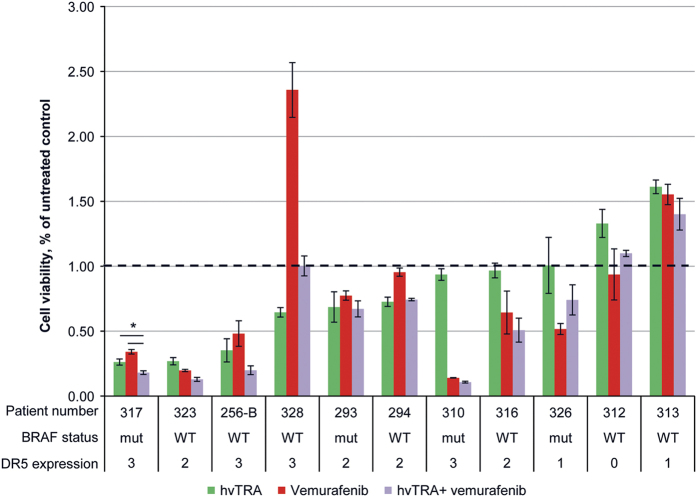
Sensitivity to hvTRA, vemurafenib and the combination in patient biopsies. Eleven biopsies from lymph node metastases from melanoma patients were made into single-cell suspensions, and seeded in 96-well round-bottom plates and exposed to hvTRA (5 *μ*g/ml), vemurafenib (5 *μ*M) or the combination. Cell viability was measured after 120 h using CellTiter-Glo Luminescent assay. Three technical replicates were performed, and the error bars represent±S.D. The luminescent signal from untreated control cells from each patient sample were set to 1, indicated by the dotted line. For DR5 expression 0; not expressed, 1; 0–10% of the cells express DR5, 2; 10–50% of the cells express DR5, 3; >50% of the cells express DR5. Patient biopsies 294 and 328 have mutated NRAS. *P<0.05.

To investigate the clinical relevance of hvTRA alone or in combination with vemurafenib, biopsies from lymph node metastases from 11 melanoma patients were collected, cultured as spheroids *in vitro* and treated with hvTRA (5 *μ*g/ml), vemurafenib (5 *μ*M) or the combination (Figure 7). The high concentrations were chosen to ensure detection of response if there was any. hvTRA reduced cell survival in six out of eleven patient biopsies (27–74% reduction), and increased the viability in two. DR5 levels were investigated by IHC, and the results showed that DR5 expression was required, but did not guarantee a response (Figure 7). Vemurafenib reduced the viability in seven of the eleven investigated patient biopsies, four with and three without the V600E/K mutation. Five patient biopsies exposed to combination therapy showed a tendency to decreased cell viability compared with the mono treatments, although it was only significant for patient number 317. In the remaining biopsies, cell survival after combination treatment was similar or higher than either mono treatments (Figure 7). These results suggest that hvTRA alone or in combination could be beneficial for a subgroup of patients; however, biomarkers are desirable to be able to predict which patients will respond.

## Discussion

It is well accepted that TRAIL induces apoptosis more efficiently in cancer cells than normal cells,^3,4^ and as a consequence different TRAs have been developed. hvTRA is a novel TRA, which has previously shown efficacy in different cancer cell lines *in vitro* and *in vivo*,^20^ but has not been studied in malignant melanoma previously. In the present study, we have investigated the efficacy of hvTRA in melanoma models of different complexity. We confirmed hvTRA-induced growth inhibition and apoptosis induction *in vitro*, and demonstrated reduced lung tissue colonization in the *ex vivo* pulmonary metastasis assay. The cell line Melmet 5 showed a modest response to hvTRA *in vitro*, but when grown as xenografts, hvTRA showed a strong and sustained growth inhibitory effect. The Melmet 5 xenografts regressed in size in all four *in vivo* experiments performed, demonstrating consistent efficacy and reproducible effect of hvTRA. An increase in DR5 protein level was observed in response to hvTRA treatment and may contribute to strengthen the already existing initial growth inhibitory effect. Furthermore, hvTRA showed efficacy in a substantial fraction of melanoma biopsies grown as spheroids *ex vivo*, implying potency of hvTRA, at least in subgroups of melanoma patients.

Different strategies have been applied to increase the efficacy of TRAs. Increasing DR5 expression^39,40^ or manipulating the apoptosis mechanisms^30,41^ leading to activation of both the extrinsic and intrinsic apoptotic pathways are two strategies. *In vitro* studies evaluating the combination of MAPK/ERK inhibitors and TRAs have been published with conflicting conclusions. Berger *et al.*^21^ show that BRAF inhibition can overcome resistance to TRAIL-induced apoptosis, while Oh *et al.*^32^ demonstrated that BRAF inhibition downregulates DR5 *in vitro*, and thereby inhibited TRAs. Our data indicate that hvTRA induces apoptosis through the extrinsic apoptotic pathway, but inhibit apoptosis activation through the intrinsic pathway by increasing the anti-apoptotic and reducing the pro-apoptotic proteins involved in the regulation of this pathway. However, when hvTRA is combined with vemurafenib the effect on the intrinsic regulating proteins was counteracted and thereby, the cells should be more primed for apoptosis induction. A strong, initial reduction in tumor volume show that the combination is potent, but the effect is not sustained, which can be related to a vemurafenib-induced reduction in DR5 expression. The limited effect of hvTRA in combination with vemurafenib is also demonstrated by reduced cleavage of pro-caspases 3 and 8 and Bid. c-jun and CHOP are transcription factors involved in regulation of DR5,^37^ and regulated by the MAPK/ERK pathway. An increase in p-c-jun was observed in response to hvTRA, while a decrease was observed in all samples treated with vemurafenib, corresponding well with the observed increase and decrease in DR5 levels. We also observed a downregulation of DR5 *in vitro*, but at vemurafenib concentrations higher than what was applied in the combination experiments.

Based on our observation and previously published data showing transient reduction in DR5 expression after MAPK/ERK inhibition,^32^ additional *in vivo* experiments with altered administration of vemurafenib were performed. Although DR5 expression was less effected with reduced vemurafenib dosing, a prolonged cytotoxic effect of the combination was not observed. The combination did induce an initial reduction in tumor volume exceeding the effect of hvTRA alone, but it was not sustained and addition of vemurafenib did not increase the growth-inhibiting effects of hvTRA in long-term experiments, suggesting that continuous dosing of vemurafenib and TRAs is not a desirable combination.

Vemurafenib usually has a very good initial effect in mutated BRAF patients, but the majority develops resistance within months. There is therefore a need to identify means to avoid or delay acquired resistance, and also alternative treatment options for patients having developed resistance and not eligible to receive immune therapy. Our results open for further investigation of TRA given on first day of vemurafenib treatment. This might, as demonstrated in the present work, enhance the growth-inhibiting effect, which could alter the surviving tumor cell population and possibly affect the progression-free survival of the patients.

Furthermore, hvTRA should be considered as a mono treatment for subgroups of melanoma patients. It has previously been shown that cells with mutated BRAF express higher DR5 levels, and respond better to DR5 targeting therapy than cells with wild-type BRAF.^37^ This implies that TRAIL receptor agonists could be a valuable option for patients with mutated BRAF, and should be evaluated in patients with acquired resistance against vemurafenib as they may presumably have high DR5 levels. In our *ex vivo* experiments utilizing patient tissue, DR5 expression was necessary, but did not guarantee response to hvTRA, implying that additional predictive biomarkers would be valuable to identify responders. We did not observe an association between mutated BRAF and DR5 levels, but the number of patients was low and a more thorough investigation on the correlation between mutation status and receptor expression is needed before any conclusion can be made. Furthermore, our patients were only tested for the most common mutations of BRAF, V600E/K, and not the more rare mutations V600D/R also responsive to vemurafenib.^42^ Thus, it is possible that some of the samples annotated as wild type may have mutations explaining the response to vemurafenib.

Despite the new drugs approved for metastatic melanoma in recent years, new treatment modalities are needed as the available options only show efficacy in a subgroup of patients and development of resistance is a significant problem. The data presented herein show that hvTRA can induce cell death *in vitro* and efficiently inhibit tumor growth *in vivo*. Synergistic or additive effects were observed when hvTRA was combined with vemurafenib *in vitro*, while reduced DR5 expression hampered the effect *in vivo*. This is valuable new information on the efficacy of hvTRA in melanoma and, to our knowledge, the first study where a TRA has been combined with a MAPK/ERK inhibitor *in vivo.* Additional studies are warranted to conclude if it is possible to take advantage of the cytotoxic effect of combining hvTRA and vemurafenib before vemurafenib-mediated downregulation of DR5. Furthermore, hvTRA as mono therapy should be evaluated more thoroughly to identify predictive biomarkers for selecting subgroups of patients for whom hvTRA will show efficacy.

## Materials and Methods

### Cell lines and culture conditions

The malignant melanoma cell lines Melmet 5 and LOX were established from lymph node metastases, while Melmet 1 was established from a subcutaneous metastasis, surgically removed at the Norwegian Radium Hospital (Oslo University Hospital, Norway) as described previously.^43,44^ The cell lines Patient-3-pre and Patient-3-post were a kind gift from Professor Peter Hersey (Royal North Shore Hospital, Sydney, Australia), and established as described previously.^45^ WM1366 was kindly provided by Professor Meenhard Herlyn (the Wistar institute, Philadelphia, PA, USA), whereas the A375 cell line was obtained from the American Type Culture Collection (Rockville, MD, USA). Green fluorescent protein (GFP) and luciferase (Luc) expressing Melmet 5 cells were generated by transducing the cells with lentivirus carrying a human ferritin promoter-driven GFP-Luc construct (described previously^46^) and kindly provided by Dr. Glen Merlino (NIH, MD, USA). The cells were grown in RPMI-1640 medium (Sigma-Aldrich, St. Louis, MO, USA), supplemented with 10% fetal bovine serum (Sigma-Aldrich) and 1% GlutaMax (Life Technologies, Carlsbad, CA, USA) at 37 °C with 5% CO_2_.

### Chemicals and drugs

The mutated RAF inhibitor vemurafenib (S1267) was purchased from Selleck (Selleck Chemicals, Houston, TX, USA), while the TRA hvTRA was a kind gift from Dr. Oliver Hill, Apogenix, Heidelberg, Germany. The hvTRA Fc-fusion protein was produced as described recently.^20^ For *in vitro* experiments vemurafenib was dissolved in DMSO, and then further diluted with RMPI-1640 medium. For *in vivo* experiments vemurafenib was dissolved in DMSO to a concentration of 50 mg/ml, and then further diluted in 0.5% methylcellulose. hvTRA was diluted in PBS for *in vivo* experiments and in RPMI-1640 for *in vitro* experiments.

### Cell viability

Cell viability after 2D culturing was measured using CellTiter 96 Aqueous One Solution (MTS; Promega, Madison, WI, USA). The cells were seeded in 96-well plates (Thermo Fisher Scientific, Rockford, IL, USA). The next day vemurafenib and hvTRA at concentrations indicated in the figure legends were added. The cells were incubated for 72 h before addition of CellTiter 96 Aqueous One Solution. After ~2 h incubation at 37 °C the absorbance was measured at 490 nm using a microplate reader (Victor^2^ 1420 Multilabel Counter, Perkin Elmer, Waltham, MA, USA).

Cell viability after 3D culturing was measured using CellTiter-Glo Luminescent Cell Viability Assay (Promega). Briefly, the cells were cultured in ultra-low attachment round-bottom 96-well plates (3000 cells/well; Sigma-Aldrich); Melmet 1 was seeded in RPMI-1640 medium containing 2% Matrigel (BD Bioscience, San Jose, CA, USA), while Melmet 5 were cultured without Matrigel. After 4 days of cultivation, hvTRA and/or vemurafenib were added at concentrations indicated in the figure legends. Following 96 h treatment viability was assessed using CellTiter-Glo Luminescent Cell Viability Assay as described by the manufacturer. Luminescence was measured using a microplate reader (Victor^2^ 1420 Multilabel Counter, Perkin Elmer). Cell viability is reported as percentage viable cells in treated as compared to untreated control samples. Both 2D and 3D experiments were performed in quadruplicates, and repeated at least three times.

### Calcusyn analysis

The Chou and Talalay CI was used to determine possible synergism. Synergistic effect is defined as CI values <1, antagonistic effect as CI values >1 and additive effect as CI=1.^47^ The CI values are calculated using Calcusyn software (BioSoft, Ferguson, MO, USA).

### Pulmonary metastasis assay

The pulmonary metastasis assay (PUMA) was performed as described earlier.^33^ GFP-Luc labeled Melmet 5 cells (5×10^5^) were injected intravenously (i.v). Fifteen minutes thereafter the mice were placed under sevofluran anesthesia (5%) and given a lethal injection of pentobarbital intraperitoneally. Trachea and lungs were exposed, and a 24 G Neoflon was inserted in the trachea under sterile conditions. The cell culture media M199 2X (Sigma-Aldrich), supplemented with 2 *μ*g/ml crystalline bovine insulin, 0.2 *μ*g/ml hydrocortison, 1 *μ*g/ml retinyl acetat and 200 U/ml penicillin/streptomycin was mixed 1 : 1 with low melting agarose (1.2%; Thermo Fisher Scientific), and slowly injected into the lungs. When lungs were completely dilated, the trachea was closed off with a 5.0 suture (Polysorb, Covidien, Dublin, Ireland). The lungs were cut out and placed in cold PBS with 100 U/ml penicillin/streptomycin. After 20 min, they were divided into 1 mm-thick slices using a Manual digital tissue chopper (Leica Biosystems, Nussloch, Germany), and placed on a gel foam (Pfizer, New York, NY, USA), which had been soaked in culture media M199 (1×; Thermo Fisher Scientific), supplemented with 1 *μ*g/ml crystalline bovine insulin, 0.1 *μ*g/ml hydrocortison, 0.5 *μ*g/ml retinyl acetat and 100 U/ml penicillin/streptomycin, for 2 h. The slices were then exposed to either just media (control) or 0.25 *μ*g/ml hvTRA. The slices were cultured at 37 °C, 5% CO_2_ for 2 weeks. The medium was changed every second day, and the lung slices were then turned over. The fluorescent microscope Olympus IX81 (Olympus, Tokyo, Japan) was used for imaging, and ImageJ (National Institute of Health, MD, USA) was used to quantify the intensity of the colonies. The settings on the microscope were changed each day pictures were taken and the intensity of treated samples can therefore only be compared with pictures of controls taken at the same day. A threshold value was set, and only cells with intensity over the threshold were chosen by the program ImageJ for further calculation. Intensity is reported as intensity in treated slices relative to untreated slices. The experiments were repeated four times, each with four technical replicates.

### *In vivo* studies

All procedures and experiments involving animals were approved by the National Animal Research Authority, and conducted according to the regulations of European Laboratory Animals Science Association. Female athymic nude foxn1 nu mice were bred at the local Department of Comparative Medicine, OUS, and kept in a specific pathogen-free environment. Food and water were supplied *ad libitum.*

In all, 2.5×10^6^ Melmet 5 cells were injected subcutaneously on the flank. When the tumors reached ~85 mm^3^, the mice were divided into three or four groups. If mice had two tumors the mean volume was used for statistical analysis. hvTRA (0.3 or 3 mg/kg) was given i.v., while vemurafenib was given orally (50 mg/kg). For treatment regimen see Figures 3 and 6. Controls were given 10% DMSO in 0.5% methylcellulose orally and PBS i.v. for as long as the other mice received treatment. Tumor volume was measured twice or three times a week, and calculated using the formula: width^2^×length×0.5. Two tumors from each group were harvested on day 1, 4 or 10, 2 h after the last treatment. Half of each tumor was snap-frozen in N_2_ for western immunoblot analysis while the other half was fixed in 10% formalin for immunohistochemical analyses. At the end of the experiments the mice were euthanized by dislocation of the neck.

### Western immunoblot analysis

Fresh frozen tumors from mice were crushed to powder using mortar and pistil. Both tumors and cells harvested from *in vitro* experiments were lysed in buffer (20 mM Tris-HCl pH 7.5, 137 mM NaCl, 100 mM NaF, 10% glycerol and 1% NP-40 supplemented with cOmplete Mini and PhosSTOP (both from Roche, Basel, Switzerland) for 1 h on ice with vortexing every 15 min before sonication. Samples were centrifuged and the supernatant transferred to new tubes and stored at −70 °C. 10–50 *μ*g protein were separated using NuPAGE novex Bis-Tris 4–12% gel (Life Technologies) and thereafter transferred to a nitrocellulose membrane using the iBlot Dry blotting system (Thermo Fisher Scientific). Membranes were blocked in either 5% dry milk or 5% BSA in TBST (0.5% Tween 20) for 1 h and incubated overnight at 4 °C with primary antibodies. Antibodies used were: DR5 (#3696), pERK (#9102), pBad^ser112^ (#4366), Bad (#9292), Bid (#2002), Bim (#2933), caspase 3 (#9664), cl. caspase 3 (#9662), PARP (#9542), Mcl-1 (#4572), CHOP (#5554), p-c-jun (#3270), Histone 3 (H3) (#4499), (all from Cell Signaling Technology, Danvers, MA, USA), caspase 8 (ALX-804-242; Enzo Life Sciences, Farmingdale, NY, USA) *α*-tubulin (#CP06; Merck Millipore, Billerica, MA, USA) and DR4 (ab#8414; Abcam, Cambridge, UK). Following primary hybridization, membranes were washed 3×10 min in TBST before applying appropriate HRP-conjugated secondary antibody for 1 h at room temperature. Membranes were then washed for 3×10 min. The signals were developed with SuperSignal West Dura Extended Duration Substrate (Thermo Fisher Scientific) and visualized in G:BOX (Syngene, Cambridge, UK).

### Immunohistochemistry

Formalin-fixed tumors were dehydrated and embedded in paraffin. Subsequently, 3-*μ*m tumor sections were prepared and placed on a microscope slide. Prior to incubation with primary antibody, paraffin sections were dewaxed in xylene and rehydrated in ethanol/water. For antigen retrieval, the tumor sections were treated at 99 °C for 25 min in citrate buffer (Target Retrieval Solution, pH 6.0, DAKO, Glostrup, Denmark). The following primary antibody was used: rabbit anti-cleaved caspase 3 (BD Biosciences). The tumor sections were incubated with the primary rabbit antibody diluted 1 : 100 in blocking buffer (PBS+20 mg/ml BSA+1 mg/ml human IgG) for 60 min at room temperature. After a PBS washing step, specific binding of the primary antibody was visualized using an anti-rabbit biotinylated secondary antibody (Southern Biotech, Birmingham, AL, USA) and streptavidin alkaline phosphatase (BioGenex, Fremont, CA, USA). The FAST-Red substrate system (DAKO) was used as the substrate for the alkaline phosphatase, which produced a red precipitate at antibody-binding sites. Sections were then counterstained with Mayer’s hematoxylin and mounted with glycerin-gelatin. A rabbit isotype control antibody was used for control staining.

### *Ex vivo* patient samples

All patients included in the study got relevant information and have signed a written consent. The study was approved by the Regional Ethical Committee (approval no: 2012/2309). Freshly operated tumor tissue from lymph node metastases was mechanically disaggregated and treated with collagenase (700 U/ml; Worthington Biochemical Corporation, Lakewood, NJ, USA) overnight at 37 °C in 5% CO_2_. The cell suspension was filtered through a 100 *μ*m nylon cell strainer (BD-Falcon, Franklin Lakes, NJ, USA) to remove debris and large cell clumps. If required, red blood cells were removed by treating the cell suspension with ACK lysis buffer according to the manufacturer’s instruction (Lonza, Walkersville, MD, USA). The cells were washed in cold PBS and resuspended in RPMI-1640 medium supplemented with 5% FCS, 2 mM L-glutamine, and 50 U/ml each of penicillin/streptavidin (the latters from Lonza, Ververiers, Belgium). Approximately 20 000 live cells, assessed by trypan blue exclusion, was seeded in round-bottom 96-well ultra-low adhesion plates in the presence/absence of hvTRA and vemurafenib alone and in combination. Effect on viability was examined using the CellTiter-Glo Luminescent Cell Viability Assay as previously described after 5 days. RT-PCR were used to decide the BRAF mutation status of the patient material. The test only detects the mutations V600E and V600K, and does not differentiate between these two mutations.

### Statistical analysis

One-Way ANOVA Tukey HSD tests were used for statistical analyses, which were performed using IBM SPSS Statistics 21 (IBM, Anmork, NY, USA). Differences were considered statistically significant if *P*-values were below 0.05, and are indicated by * in the figures.
