# Supplementary figures and images for: hvTRA, a novel TRAIL receptor agonist, induces apoptosis and sustained growth retardation in melanoma
Source: Cell Death Discov. 2016 Dec 12;2:16081–. doi: 10.1038/cddiscovery.2016.81 (PMC5149582; doi:10.1038/cddiscovery.2016.81)

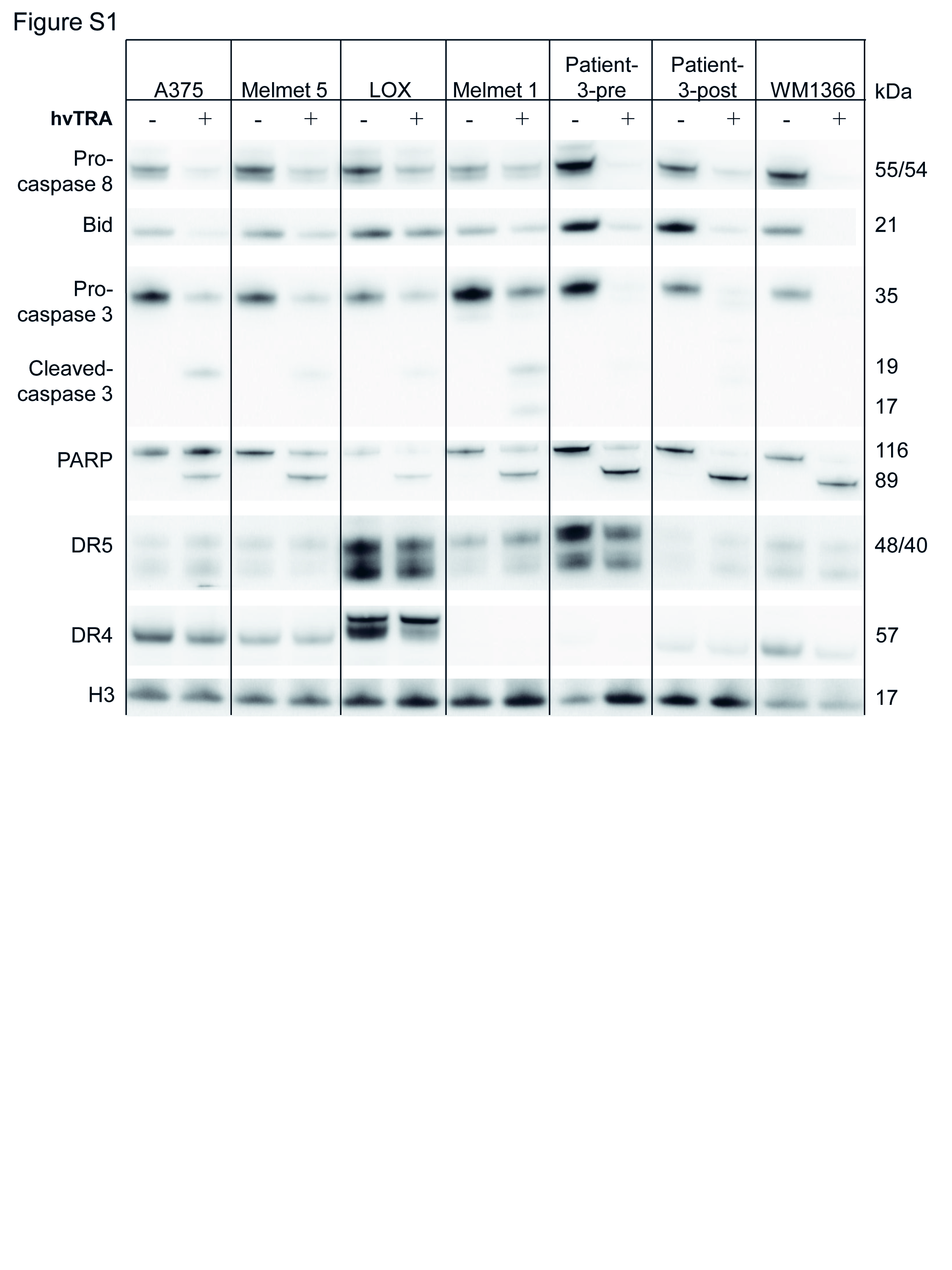

Supplement: Supplementary Figure S1 [file cddiscovery201681-s1.tiff]

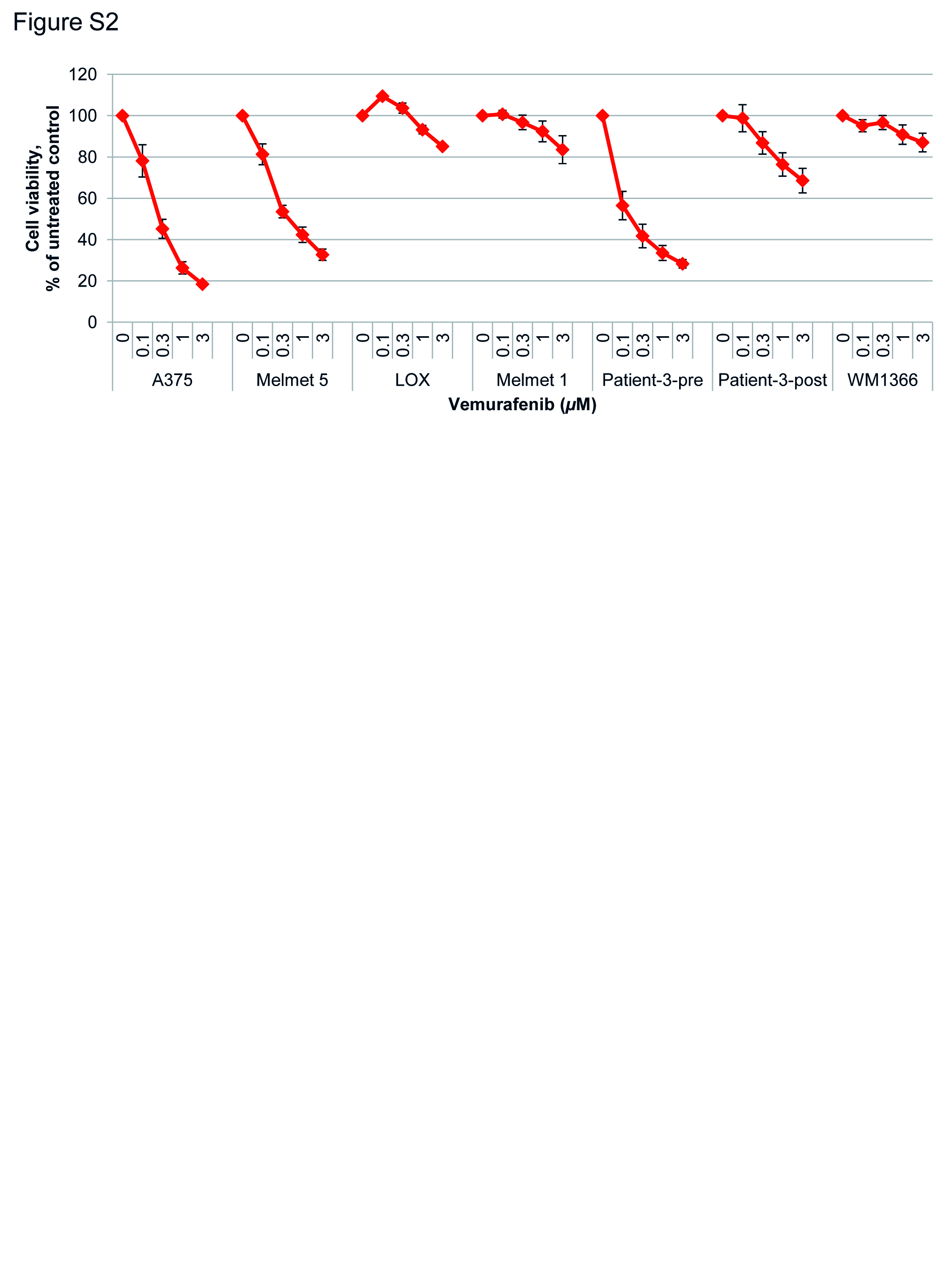

Supplement: Supplementary Figure S2 [file cddiscovery201681-s2.tiff]

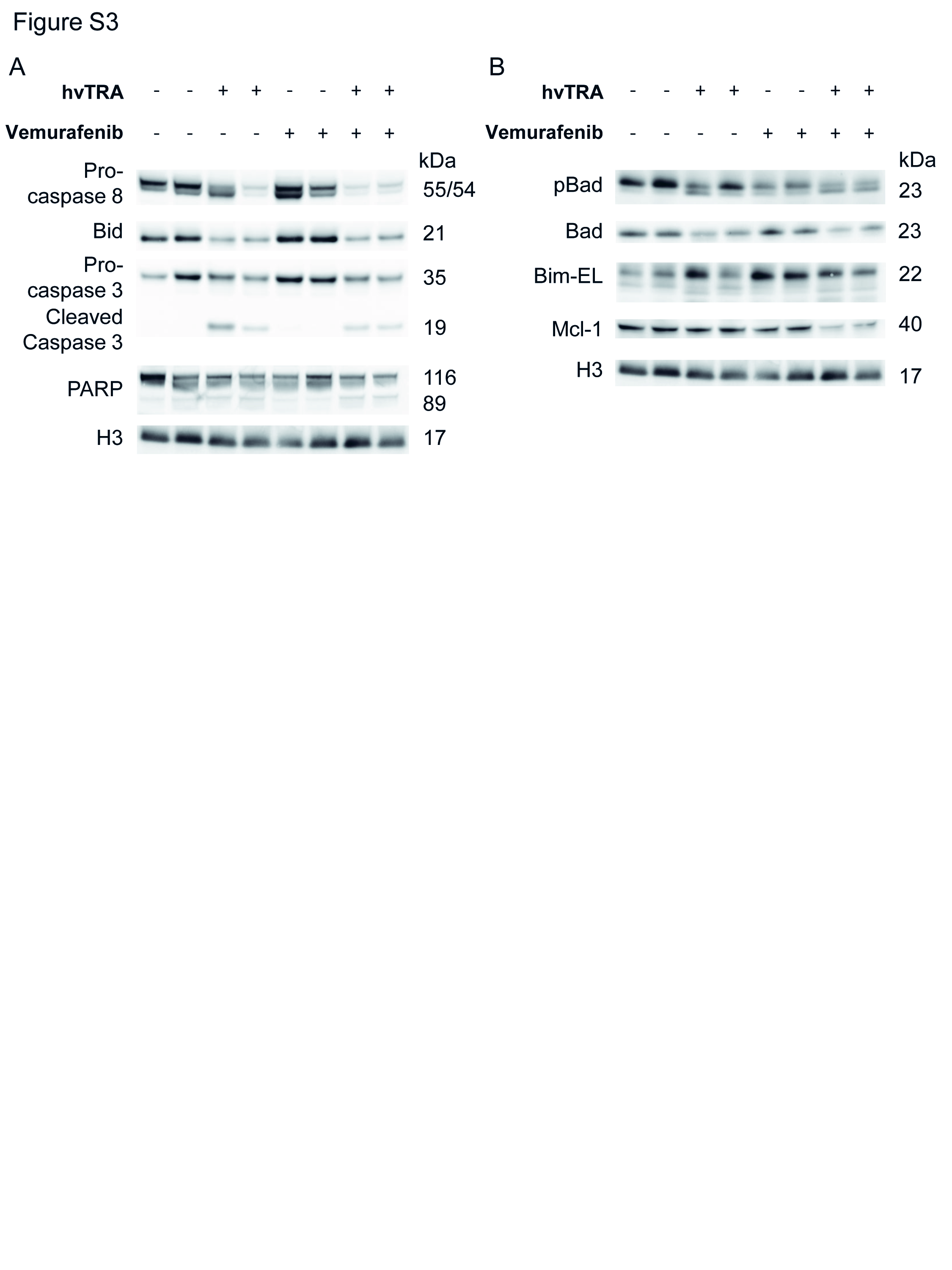

Supplement: Supplementary Figure S3 [file cddiscovery201681-s3.tiff]

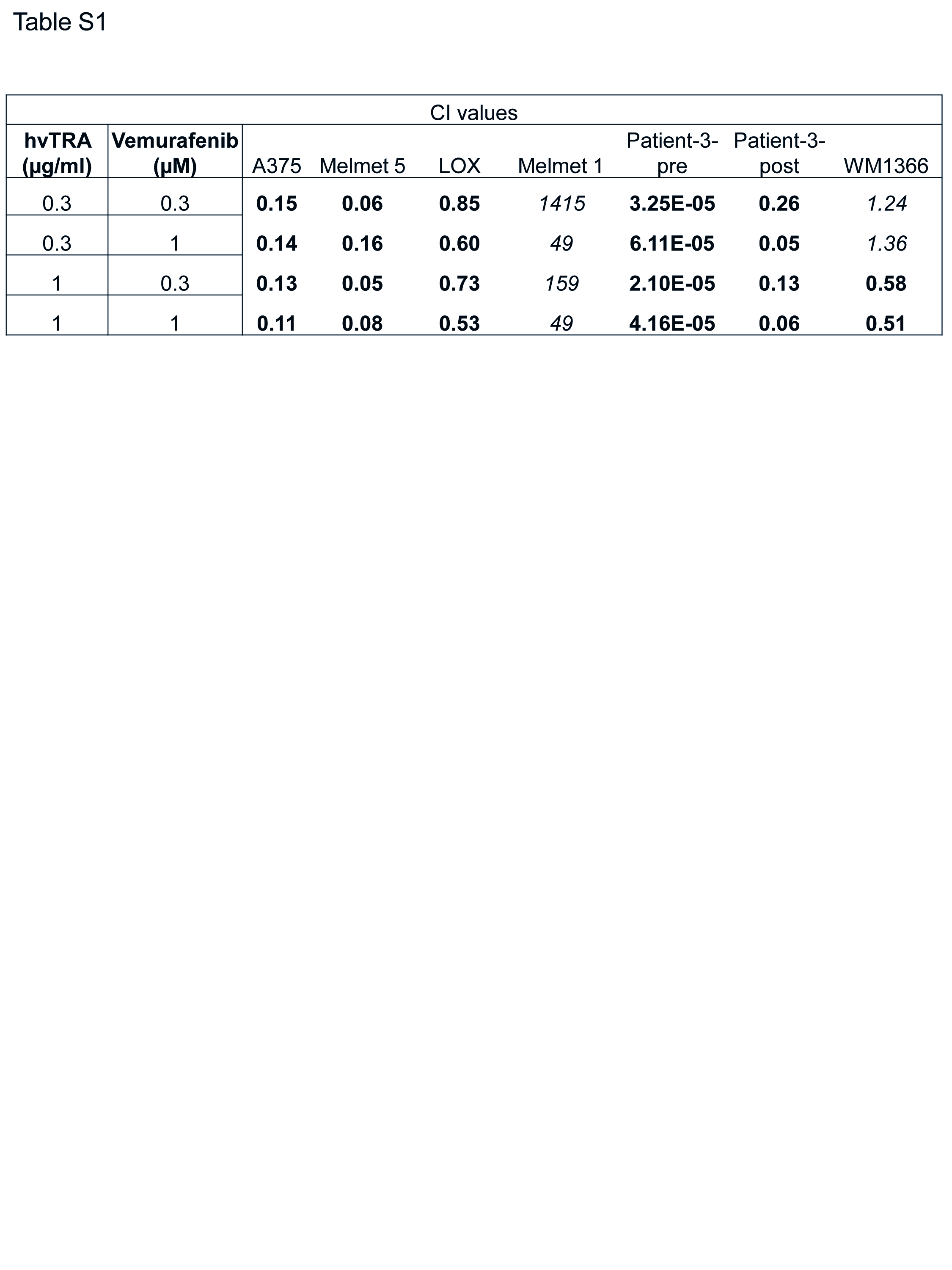

Supplement: Supplementary Table S1 [file cddiscovery201681-s4.tiff]
